# Automated quantification of Ki-67 proliferative index of excised neuroendocrine tumors of the lung

**DOI:** 10.1186/s13000-014-0174-z

**Published:** 2014-10-16

**Authors:** Sandy Z Liu, Paul N Staats, Lindsay Goicochea, Borislav A Alexiev, Nirav Shah, Renee Dixon, Allen P Burke

**Affiliations:** From the Departments of Pathology and Internal Medicine, University of Maryland, 22 S. Greene St, Baltimore, 21201 USA

**Keywords:** Proliferation index, MIB-1, Ki-67, Carcinoid tumor, Neuroendocrine carcinoma, Lung neoplasms, Pathology

## Abstract

**Background:**

The histopathologic distinction between typical carcinoid (TC) and atypical carcinoid (AC) of the lung is based largely on mitotic index. Ki-67 may aid in separation of these tumors, as well as the distinction from large cell neuroendocrine carcinoma (LCNEC).

**Methods:**

We identified 55 surgically resected primary neuroendocrine lung tumors (39 TC, 7 AC, 9 LCNEC) based on mitotic rate and histologic features. Ki-67 proliferative index based on automated image analysis, tumor necrosis, nodal metastases, local or distant recurrence, and survival were compared across groups.

**Results:**

The mean mitotic count and Ki-67 index for TC, AC, and LCNEC were 0.1 and 2.3%, 3.4 and 16.8%, and 56.1 and 81.3% respectively. The Ki-67 index did not overlap among groups, with ranges of 0–6.7% for TC, 9.9-25.7% for AC, and 63.2-91.9% for LCNEC. Nodal metastases were identified in 4/39 (10%) TC, 2/7 (22%) AC, and 2/8 (25%) LCNEC. There was no survival difference between TC and AC, but there was a significant survival difference between LCNEC and TC and AC combined (p < 0.001). There was a step-wise increase in disease free survival with tumor grade: no TC recurred, 2/7 AC recurred or progressed (median interval 35.5 months), and all LCNEC recurred or progressed (median interval 10.1 months). No patient with TC or AC died of disease, compared to 7/8 LCNEC with follow-up data.

**Conclusions:**

We conclude that Ki-67 index is a useful diagnostic marker for neuroendocrine tumors, with 7% a divider between AC and TC, and 50% a divider between LCNEC and AC. LCNEC is biologically different from AC and TC, with a much more aggressive course, and a high Ki-67 index.

**Virtual Slides:**

The virtual slide(s) for this article can be found here: http://www.diagnosticpathology.diagnomx.eu/vs/13000_2014_174

## Background

Neuroendocrine tumors of the lung account for approximately 20-25% of primary lung tumors [1]. The most common type is small cell carcinoma, accounting for 15-20%, followed by large cell neuroendocrine carcinoma (LCNEC) (~3%), typical carcinoid (TC), and atypical carcinoid (AC) tumors (~1-2%). Other than small cell carcinomas, neuroendocrine tumors are typically initially treated by surgical excision. The distinction between these four tumor types is based on histologic features, mitotic index, and presence or absence of necrosis [1,2]. Of these features, mitotic figures are particularly important in separating AC from TC (0–1 mitotic figures in 10 high-power microscopic fields (HPF) for TC, 1–10 mitotic figures/10 HPF for AC, and >10/10 mitotic figures/10 HPF for LCNEC).

Despite diagnostic criteria, inter-observer variability exists between typical and atypical carcinoid tumors [3,4]. Furthermore, diagnostic challenges can occur in a biopsy due to limited sampling or poor specimen handling (crush artifact) [5]. A distinction is important because of the different prognosis and treatment of carcinoid tumors vs. high-grade neuroendocrine carcinomas [6,7]. Several studies have shown a correlation between a high Ki-67 and a poorer prognosis [8-14]. Ki-67 has been shown to be more reliable and reproducible in distinguishing TC from AC than histology [3]. Additionally, a very high Ki-67 index can help distinguish LCNEC from AC when classification is doubt.

While previous investigations have correlated clinicopathologic characteristics and Ki-67 index in carcinoid tumors, relatively few studies have studied the spectrum of TC, AC, and LCNEC and provided diagnostic numeric criteria using Ki-67 similar to mitotic index. The purpose of this study is to correlate Ki-67 mitotic index calculated by digital image analysis with clinicopathologic variables of non-small cell neuroendocrine tumors and to provide specific ranges of proliferative index for diagnostic use.

## Methods

### Study population

A search of electronic pathology database with the key words “carcinoid”, “large cell neuroendocrine”, and “neuroendocrine” of surgically resected lung tumors (wedge resection, lobectomy, pneumonectomy, airway resection) from January 2003 to December 2014, inclusive, revealed a total of 62 cases originally diagnosed as primary non-small cell neuroendocrine tumors. The study only included resection specimens; no biopsies were included. Secondary, recurrent, and metastatic tumors were also excluded. One tumor originally diagnosed as “poorly differentiated adenocarcinoma with neuroendocrine features” was reclassified as large cell neuroendocrine tumor based on the most recent World Health Organization criteria. One tumor originally diagnosed as “high grade neuroendocrine tumor” was reclassified as small cell carcinoma and excluded. Six tumors were excluded because of lack of histological material.

### Pathology and histological classification

All cases were reviewed by at least 2 study pathologists to confirm their classification (ABP, SZL) based on the current WHO criteria for lung neuroendocrine tumors. TC was defined as well-differentiated neuroendocrine tumor with 0–1 mitoses/10 HPF, and without necrosis. ACs were distinguished by 2–10 mitoses/10 HPF and/or focal necrosis. LCNECs had > 10 mitotic figures in 10 HPF, usually with large areas of necrosis; showed neuroendocrine morphology (nuclear palisading with nests, rosette-like, or ribbons of cells); had prominent nucleoli and cytoplasm unlike small cell carcinoma; and showed immunohistochemical evidence of neuroendocrine differentiation (diffuse staining for either synaptophysin, chromogranin, or CD56 [15]. All tumors stained positive for at least one of three neuroendocrine markers: synaptophysin, chromogranin, or CD56.

The tumor size was obtained from the gross description in the surgical pathology laboratory. The tumor location was obtained from clinical and radiological information. Immunohistochemical staining was performed using an automated immunostainer (BenchMark, Ventana, Tucson, AZ) and Ultraview universal indirect biotin-free DAB detection kit. The following neuroendocrine and proliferative immunohistochemical markers were used: mouse monoclonal synaptophysin (Ventana), mouse monoclonal chromogranin-A (Ventana), mouse monoclonal CD56 (Ventana), and rabbit monoclonal Ki-67 (clone 30–9, Ventana).

### Mitotic count and Ki-67 proliferative index

The mitotic activity was manually quantitated in the most cellular areas on whole-slide images scanned using the Aperio imaging system (Leica Biosystems, Buffalo Grove, IL); four 40x high power fields were calibrated as 1 mm^2^. The mitotic count was performed according to the method recommended in the 7^th^ edition of the AJCC Cancer Staging Manual: enumerate mitoses in the most mitotically active area (“hot spot”) and then extend mitotic count to adjacent contiguous fields. If no mitotic activity is evident, random representative tumor fields are scanned for mitoses.

The Ki-67 proliferative index (PI) was performed blinded to any knowledge of mitotic counts and was quantitated using a validated nuclear algorithm (Aperio, Leica Biosystems, Buffalo Grove, IL), where slides were scanned at 20× magnification using the Aperio Scanscope Console and the images were analyzed using ImageScope Nuclear v9 algorithm software that reports the total number of nuclei counted and the percentage of positive cell nuclei. The Ki-67 digital image analysis had been previously validated for clinical and research use by comparing Aperio-generated results with scores using the Chromovision ACIS system (which had, in turn, been previously validated against manual Ki-67 counts) performed on the same slides of breast cancers. In brief, digital image analysis was completed twice for each test case using the Aperio Imagescope algorithm, and the outputs were then averaged and compared to the Chromovision ACIS results, which yielded the recommended acceptable level of agreement [16]. In our study, digital image analysis was executed by manually annotating at least three representative non-necrotic tumor fields which together contained at least total 2000 cells (or all tumor cells if less than 2000) in which automated analysis was to be performed. Positive staining was defined as faint nuclear positivity or greater (1+). Care was taken to exclude fields containing substantial numbers of non-tumor cells, such as endothelial cells and intratumoral lymphocytes, and non-tumor cells were further excluded by calibrating the algorithm thresholds for nuclear size (>10 um), nuclear radius, nuclear roundness, and curvature so as to omit non-neoplastic cells. Limitation of scoring to tumor cells was verified through visual review of the marked-up digital images and the corresponding H&E-stained scanned slide.

### Statistical analysis

All statistical analyses were performed using JMP 10.0 statistical software (SAS, USA). ANOVA analysis was performed between histological group (TC, AC, and LCNEC) and Ki-67 index. Fisher’s two-sided exact test was used to analyze categorical data (race, smoking, gender, necrosis). Overall survival was calculated from the date of pathological diagnosis to time of death or last follow-up, and disease free survival was calculated from date of pathological diagnosis to time of last clinical evidence of recurrence, progression, or death. Overall survival curves and significance of survival and disease free survival distributions were generated using Kaplan-Meier and Log-Rank method. All p-values were two-sided. Multivariate survival fit was calculated by the Weibull parametric test.

## Results

### Patient population

There were a total of 55 lung neuroendocrine tumors: 39 TC, 7 AC, and 9 LCNEC. In three patients, the neuroendocrine tumor was found incidentally: one TC discovered at wedge resection for presumed metastatic hepatocellular carcinoma, one TC discovered at pulmonary explant for emphysema, and one synchronous TC discovered at lobectomy for lung adenocarcinoma. There were a total of 41 females and 14 males (3:1 ratio),

### Clinical features

Clinical characteristics are summarized in Table 1. All patients with LCNEC were smokers compared to 18 of 37 patients with TC or AC (49%)(p = 0.0050, Fisher’s exact test, two-sided). There was a strong association with LCNEC and black race, compared to TC and AC (p = 0.0005, Fisher’s exact test, two-sided). Fifty-three tumors were M0 and 2 tumors (one LCNEC, one AC) were M1 at time of presentation. There was no association between tumor size, location, TNM stage, gender, and age with tumor histology.

**Table 1:** **Pathological characteristics**

	**All tumors**	**Typical**	**Atypical**	**Large cell**
**Mitotic count (** ^**#**^ **/10 HPF)** ^**‡**^	10.4 ± 1.5	0.1 ± 0.2	3.4 ± 2.1	56.1 ± 17.0
**Range**	0-79	0-1	2-8	25-73
**Ki-67 index ± SD (%)** ^**‡**^	17.0 ± 29.4	2.3 ± 1.8	16.8 ± 7.7	81.3 ± 9.9
**Range (%)**	0-91.9	0 - 6.7	9.9 - 25.7	63.2 - 91.9
**Presence of necrosis** ^**#**^				
***Absent***	43 (78%)	39 (0%)	4 (57%)	0 (0%)
***Present***	12 (22%)	0 (100%)	3 (43%)	9 (100%)
**Presence of LVI**				
***Absent***	43 (78%)	33 (85%)	5 (71%)	5 (56%)
***Present***	12 (22%)	6 (15%)	2 (29%)	4 (44%)
**Presence of perineural invasion**				
***Absent***	48 (87%)	36 (92%)	7 (100%)	5 (56%)
***Present***	7 (13%)	3 (8%)	0 (0%)	4 (44%)
**Spindle histology**				
***Absent***	48 (87%)	32 (82%)	5 (71%)	9 (100%)
***Present***	7 (13%)	7 (18%)	2 (29%)	0 (0%)
**Osseous metaplasia**				
***Absent***	49 (89%)	34 (87%)	6 (86%)	9 (100%)
***Present***	6 (11%)	5 (13%)	1 (14%)	0 (0%)
**Original pathological diagnosis**				
***Typical carcinoid***	40	36	4	0
***Atypical carcinoid***	8	3	3	2
***Large cell neuroendocrine***	4	0	0	4
***Small cell carcinoma***	2	0	0	2
***Poorly differentiated NSCLC***	1	0	0	1

^‡^p < 0.0001, ANOVA, across all groups.

^#^p < 0.0001, Fisher's exact test, comparing large cell NE vs. typical carcinoid.

^#^p = 0.0192, Fisher's exact test, comparing large cell NE vs. atypical carcinoid.

^#^p < 0.0001, Fisher's exact test, comparing atypical carcinoid vs. typical carcinoid.

### Histological features

**Figure 1:**
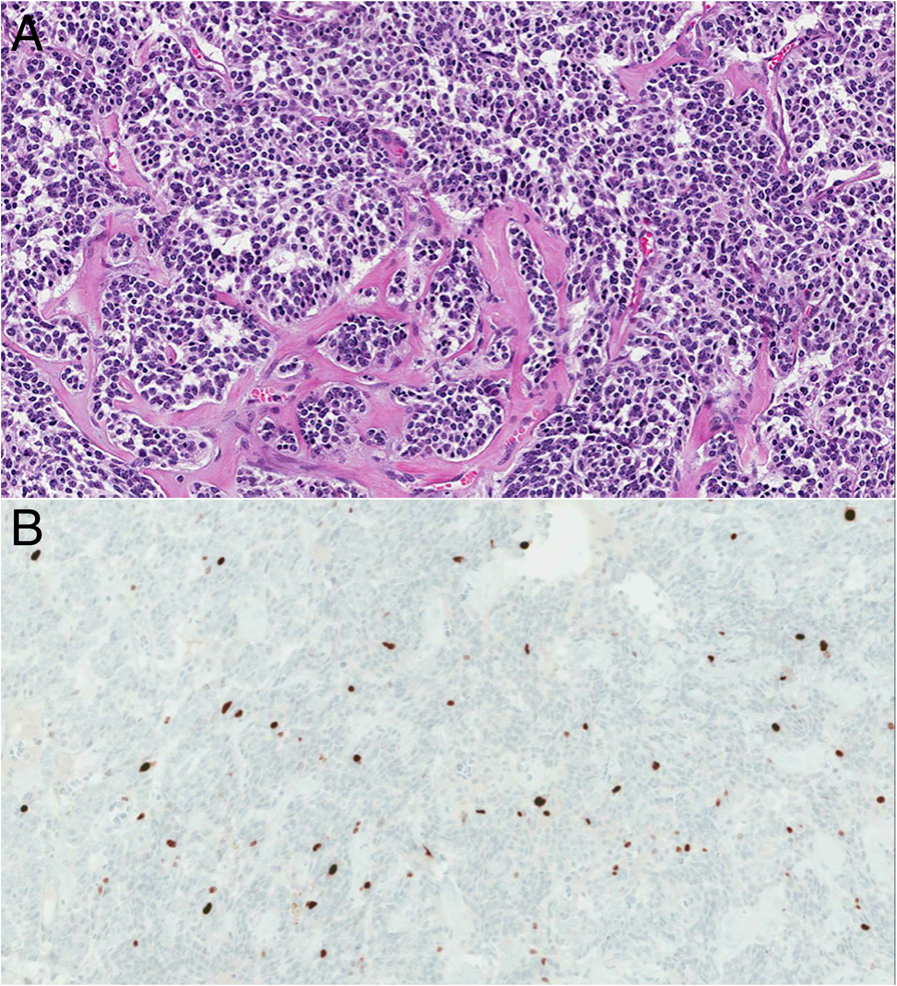
**Typical carcinoid. A**. Typical carcinoid tumor with osseous metaplasia showing anastomosing nests and cords. No mitoses or necrosis are identified. **B**. Ki-67, 20x magnification, showing 4.6% tumor positivity by digital image analysis.

**Figure 2:**
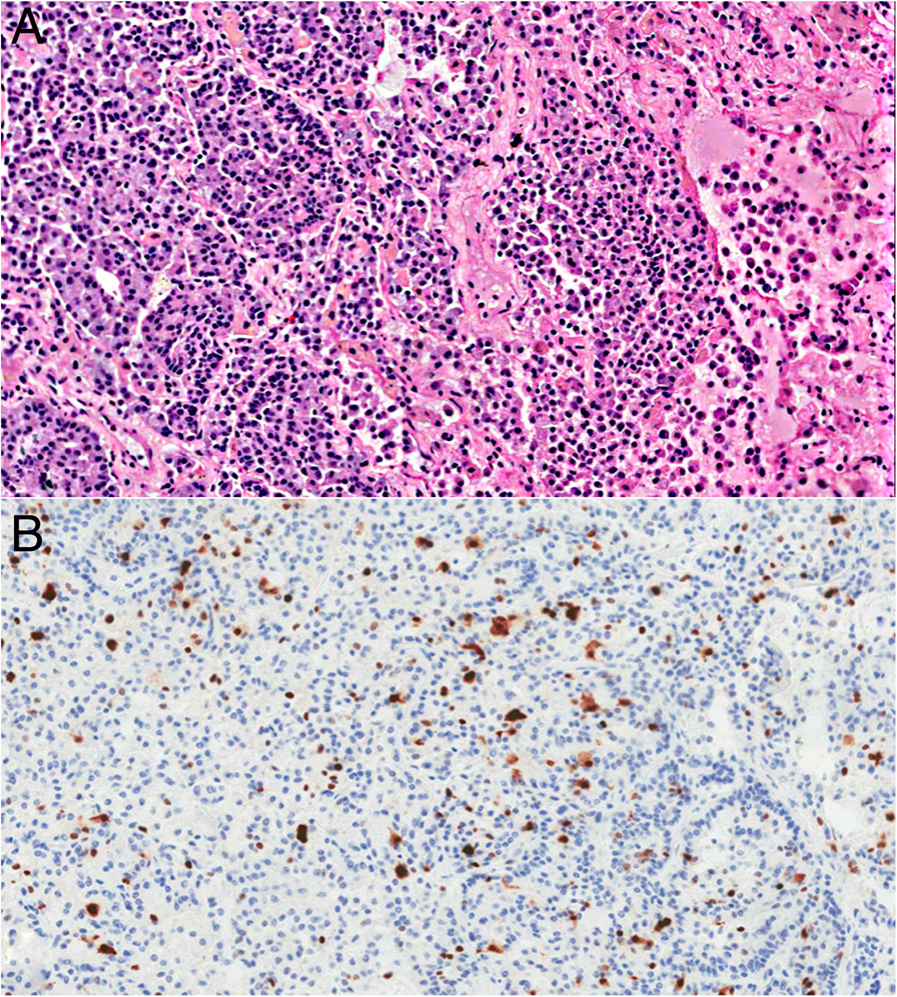
**Atypical carcinoid. A**. H&E, 20x magnification, atypical carcinoid showing a solid nested growth pattern and geographic necrosis. Mitosis is not present in this field. **B**. Ki-67, 20x magnification, showing 12.7% tumor positivity by digital image analysis.

**Figure 3:**
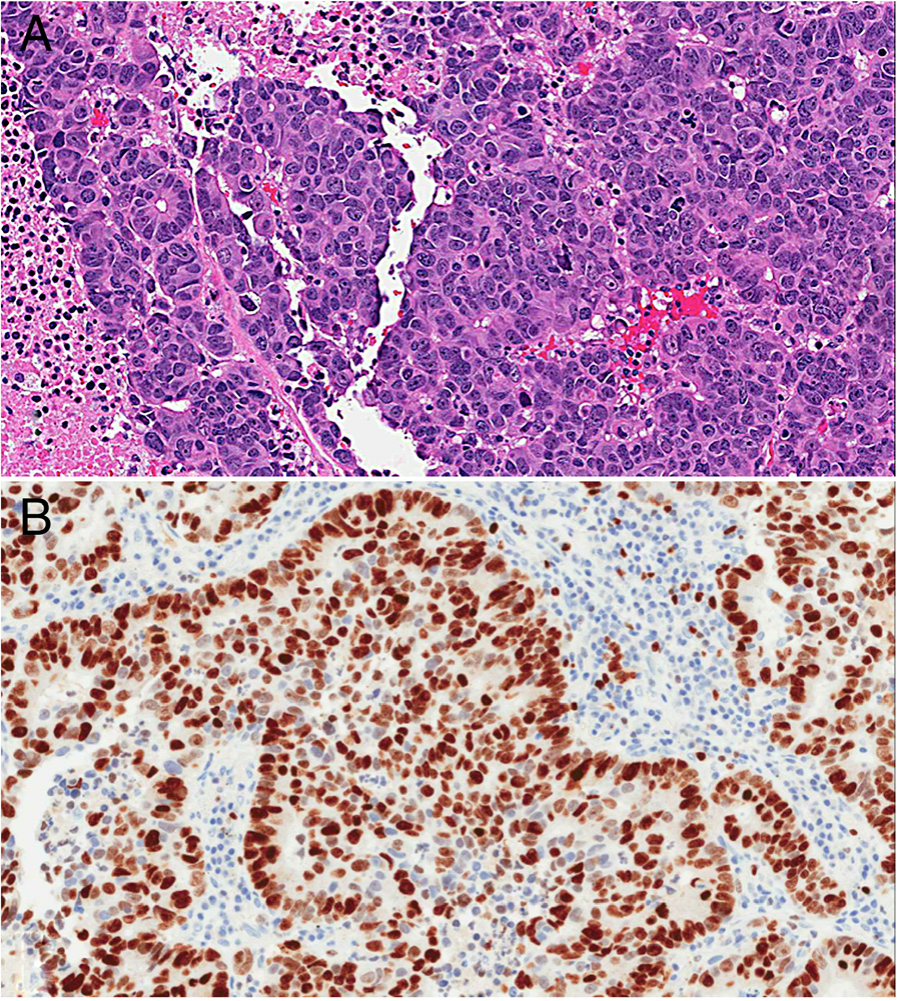
**Large cell neuroendocrine carcinoma. A**.H&E, 20x magnification, large cell neuroendocrine tumor with solid and pseudoglandular growth patterns. Prominent nucleoli, geographic necrosis, and mitoses are readily evident. **B**. Ki-67, 20x magnification, showing 91.9% tumor positivity by digital image analysis.

**Figure 4:**
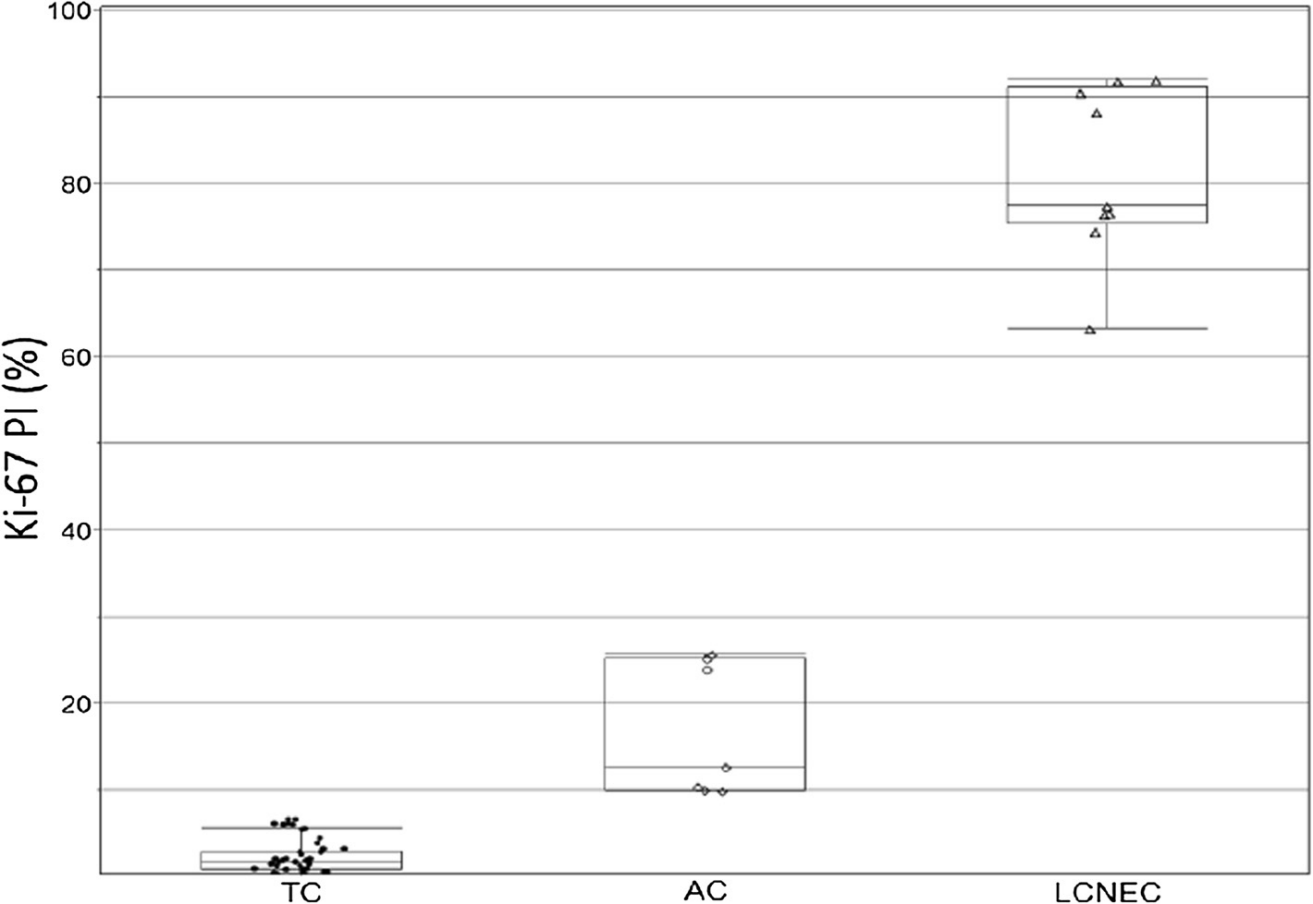
**Ki-67 proliferative index by tumor type.** Scatterplot of Ki67 index by histological type. Error bars represent standard deviation with mean. TC = typical carcinoid; AC = atypical carcinoid; LCNEC = large cell neuroendocrine carcinoma.

**Figure 5:**
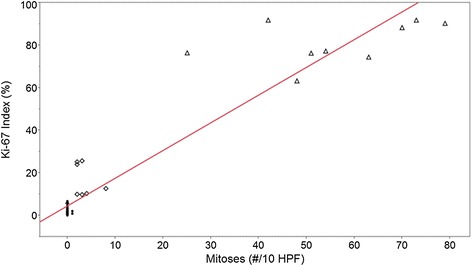
**Ki-67 proliferative index versus mitoses.** Scatterplot with simple linear regression of KI-67 PI vs. mitosis (#/10 HPF). Dot- TC, Diamond-AC, Triangle- LCNEC. KI-67 PI (%) = 4.59 + 1.30 (mitoses/# 10HPF), R^2^ = 0.89, p < 0.001.

The histological characteristics are summarized in Table 2. The ranges for mitotic count did not overlap by definition (Figures 1, 2 and 3). In addition the Ki-67 index for TC, AC, and LCNEC did not overlap (Figure 4). The mean mitotic count and Ki-67 index across all groups were statistically different (ANOVA, p < 0.0001). Among all tumors, in aggregate, there was a strong correlation between mitotic count and Ki-67 PI by linear regression (R^2^ = 0.90, p < 0.0001) (Figure 5). Necrosis, as expected, showed an increase in incidence with higher grade (Table 2). There was no significant difference in lymphovascular and perineural invasion with increasing tumor grade (Table 2). Spindled histology and osseous metaplasia were observed only in TC and AC.

**Table 2:** **Clinical characteristics**

	**All tumors**	**TC**	**AC**	**LCNEC**
**n**	55	39	7	9
**Mean age ± SD (yrs)**	60.8 ± 11.2	61.8 ± 12.6	61.5 ± 6.5	55.2 ± 5.5
**Range (yrs)**	33.6-79.4	33.6-79.4	51.1-73.4	47.5-61.6
**Sex**				
**Females**	41 (75%)	31 (80%)	5 (71%)	5 (56%)
**Males**	14 (25%)	8 (20%)	2 (29%)	4 (44%)
**Race** ^**‡**^				
***White***	41	32	6	3
***Black***	12	5	1	6
***Other***	2	2	0	0
**Smoking hx** ^**†**^				
***Smoker***	27 (59%)	16 (50%)	2 (40%)	9 (100%)
***Nonsmoker***	19 (41%)	16 (50%)	3 (60%)	0 (0%)
***Data unavailable***	9	7	2	0
**Location**				
***Central***	46 (84%)	34 (87%)	6 (86%)	6 (67%)
***Peripheral***	9 (18%)	5 (13%)	1 (14%)	3 (33%)
**Mean tumor size ± SD (cm)**	2.2 ± 1.5	2.1 ± 1.5	2.7 ± 1.9	2.8 ± 1.2
***Range (cm)***	0.2-6.9	0.2-6.9	0.7-5.5	0.4-4.5
**Tumor stage (T)**				
***T1a***	26	19	3	4
***T1b***	10	8	2	0
***T2a***	16	10	1	5
***T2b***	1	1	0	0
***T3***	2	1	1	0
**Nodal stage (N)**				
***Nx***	1	0	0	1
***N0***	46	35	5	6
***N1***	5	3	1	1
***N2***	3	1	1	1
**Metastatic stage (M)**				
***M0***	53	39	6	8
***M1***	2	0	1	1
**Procedure**				
***Other***	2	1	1	0
***Wedge***	14	5	2	7
***Segmentectomy***	2	2	0	0
***Lobectomy***	30	25	4	1
***Bilobectomy***	4	4	0	0
***Pneumonectomy***	3	2	0	1

AC = atypical carcinoid; TC = typical carcinoid; LCNEC = large cell neuroendocrine carcinoma.

^‡^p = 0.0005 Fisher's exact test, LCNEC vs. TC and AC, Whites vs. Blacks.

^†^p = .005, LCNEC vs. AC and TC.

### Survival and progression free survival analysis

**Figure 6:**
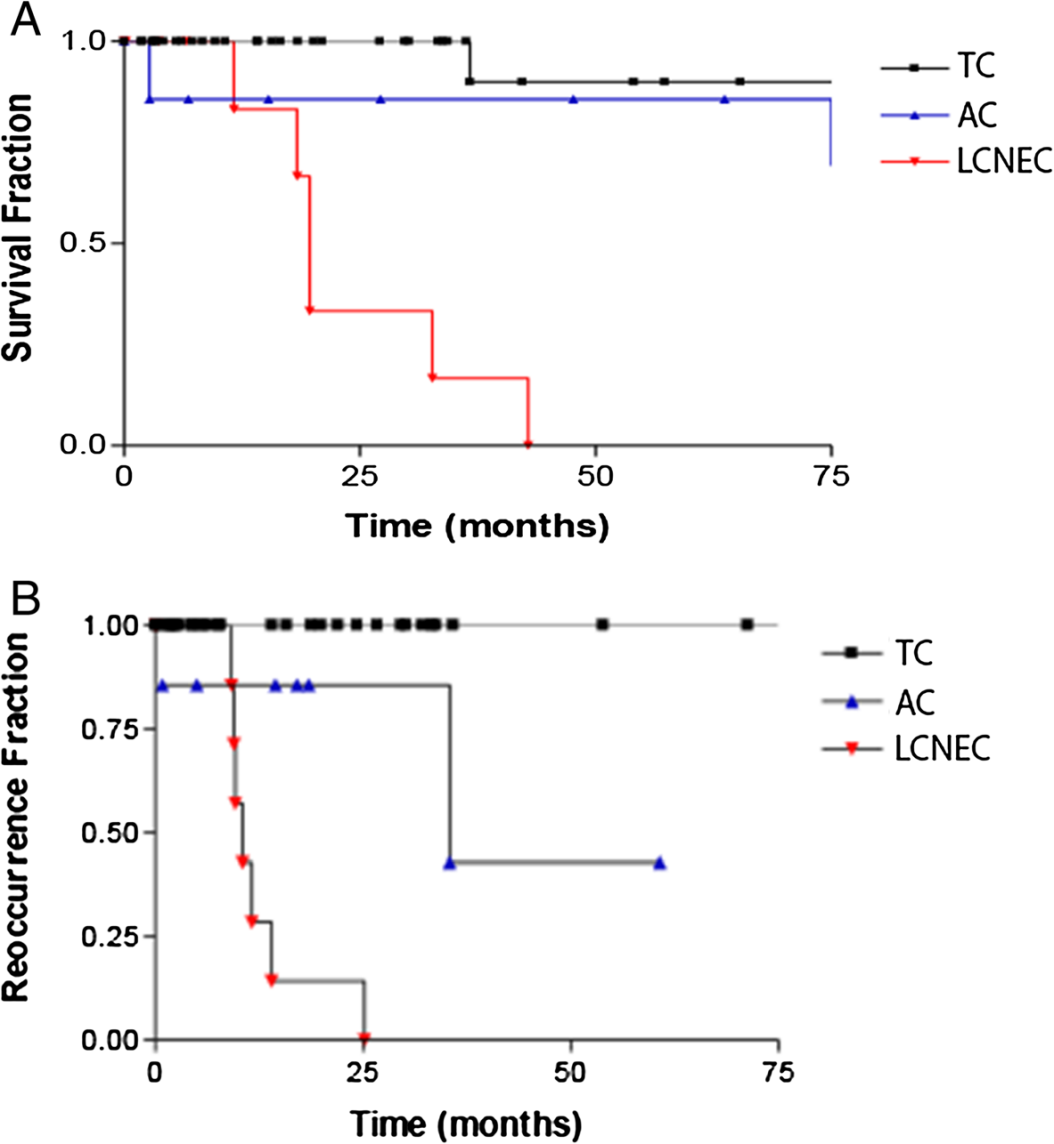
**Overall and disease free survival by tumor type. A**: Kaplan-Meier Curves, overall survival by histological type. Although there is a marked difference between LCNEC and carcinoid, there is no difference between typical and atypical carcinoid. **B**: Kaplan-Meier Curves, disease free survival by histological type. The recurrence rate for AC is intermediate between TC and LCNEC.

Survival and progression free survival analysis are summarized in Table 3. Available clinical information for disease status at last follow-up was available in 47 of 55 cases, 33 of 39 TC, 7 of 7 AC, and 8 of 9 LCNEC. At the end of the study, 11 patients were deceased: 3 with TC, 1 with AC, and 7 with LCNEC. Three patients with TC died of other causes, two of which were known: one from metastatic hepatocellular carcinoma and one from recurrent pulmonary adenocarcinoma. There was no disease recurrence in TC (0/33). Two patients with AC had progression of disease: one with metastatic nodal disease and one with distant metastases. The deceased patient with AC had metastatic disease at initial presentation, and died from disease 2.5 months after diagnosis. Seven patients with LCNEC died of disease, one patient is alive with recurrent disease,, and one patient did not have sufficient follow-up. No survival difference was observed between TC and AC. (Figure 6A). There was a step-wise decrease in disease free survival with increasing tumor grade: TC-not reached, AC- 35.5 months, LCNEC- 10.1 months (Figure 6B). TC also had a significantly improved disease free survival compared to AC (p = 0.0168). There was also step-wise decrease in median overall survival with tumor grade. The median overall survival was not reached in TC and AC and both groups had significantly improved survival compared to LCNEC (median OS- 18.3 months, p < 0.001).

**Table 3:** **Reoccurrence and survival data**

	**All tumors**	**TC**	**AC**	**LCNEC**
**Clinical status at last f/u**				
***Alive without recurrence***	35	30	5	0
***Alive with recurrence***	2	0	1	1
***Alive, unknown***	7	6	0	1
***Died with disease***	8	0	1	7
***Died of other causes***	2	2	0	0
***Died, unknown cause***	1	1	0	0
**Median disease free survival (months)** ^**†**^	NR	NR	35.5	10.1
**Hazard ratio**		<0.01	0.10	1.00
**Confidence interval (95%)**		-	0.01-0.57	-
***P-value***	*-*	**<0.0001**	**0.0063**	-
***Mean DFS follow-up (months)***	20.2	21.9	21.9	12.0
**Median survival (months)**	NR	NR	NR	19.7
**Hazard ratio**		0.06	0.08	1.00
**Confidence interval (95%)**		0.01-0.24	0.00-0.54	-
***P-value***	*-*	**<0.0001**	**0.0070**	-
***Mean OS follow-up (months)***	29.6	31.4	34.3	18.3

^†^p = 0.0168, DFS, TC vs. AC.

AC = atypical carcinoid; TC = typical carcinoid; LCNEC = large cell neuroendocrine carcinoma.

Multivariate survival analysis was performed with independent variables of age, gender, mitotic figures, and Ki-67 index, with both recurrence and death as end-points. Ki-67 proliferative index was independently associated with death (p = .005) and recurrence (p = .02). In contrast, mitotic rate was not significantly associated with death (p = .07) or recurrence (p = 0.4), nor was age (0.7 for both). Gender showed no significant association with survival or recurrence (p > 0.05).

## Discussion

There is consensus that small cell carcinomas of the lung, in addition to having a high mitotic rate, have a Ki-67 PI greater than 50% [17-20]. However, there are fewer data on Ki-67 PI regarding pulmonary TC, AC and LCNEC. Most reported studies have used manual methodologies for calculating Ki-67 PI. In these studies, the mean Ki-67 PI for TC, AC, and LCNEC were 0.5-3.7%, 2.4-20%, and 25-81% respectively [11,12,19,21-24]. There is no consensus threshold separating pulmonary AC from TC and LCNEC, with reported cutoff threshold ranging between 2.5% to 5% for TC v. AC, and a value of 30% for AC v. LCNEC [9,11,12,19]. In the gastrointestinal tract, corresponding cutoffs are 3% and 20%, using both manual and automated Ki-67 methodology [25]; Yamaguchi, 2013 #41}, separating low from intermediate grade, and intermediate grade from high-grade neuroendocrine carcinomas, respectively.

The current study is the first large quantitative study utilizing an established commercially available platform (Aperio®) to quantify the Ki-67 PI of TC, AC, and LCNEC. Our reported mean Ki-67 PI for TC, AC, and LCNEC were 2.3%, 16.8%, and 81.3% respectively. A cut-off of 7% reliably separated all TC and AC and a cutoff of 50% was a reliable cutoff between AC and LCNEC, with a wide interval of Ki-67 PI in which AC and LCNEC did not overlap (30-60%). A similar study on cytology smears, found a large range of Ki-67 PI (25-50%) where low-grade neuroendocrine and high-grade neuroendocrine tumors did not overlap [17]. Our mean Ki-67 proliferative index across the spectrum of neuroendocrine tumors is somewhat higher than in most reported studies, possibly due to our differences in methodology in our using automatic cell counting – while this method has been previously validated against manual counting in breast cancers, it may have a lower threshold for counting weakly positive cells than some observers. Indeed, one of the strengths of automated quantitation of Ki-67 PI is elimination of interobserver variability in threshold for positivity. Our data for mean Ki-67 is similar to a recent study by Watts et al., who reported a mean of 3.7% and 18.8% for TC and AC respectively [14]. However, a Ki-67 of up to 50% in two AC in two studies and up to 99% in one LCNEC have been reported [14,24,26]. In an older study by Costes et al., a Ki-67 of 0–3.0% for TC and 0–6.1% for AC was reported [9].

Determination of Ki-67 PI may be useful in small biopsies or in cytological specimens where diagnostic tissue is limited for morphologic and proliferative activity assessment due to small sample size and or crush artifact. In these samples, a carcinoid tumor maybe over-diagnosed as high-grade neuroendocrine tumor or vice versa [18,20]. Although this study was limited to resection specimens, and the proposed cutoffs require validation before use in small biopsy or cell block specimens, previous studies seem to support the value of Ki-67 PI in such specimens. Watanabe et al. found no mitotic figures in 7 of 38 small biopsies of LCNECs, and 11 of 38 small biopsies had inadequate tumor volume for mitotic count (<10 HPF); nevertheless, a Ki-67 index could adequately be assessed in all biopsies, and it was found to be in a range of 42-99% [24]. Furthermore, at least one study has shown Ki-67 PI is a reliable test in inter-observer agreement between TC and AC [3]. Accurate distinction between a high grade and low to intermediate grade neuroendocrine tumor is important because of different biological behaviors, including overall survival and disease free survival, among TC, AC, and high-grade neuroendocrine tumors [7,15,21].

Digital image analysis of Ki-67 PI has been shown to have improved diagnostic accuracy compared to visual estimation for neuroendocrine tumors of the gastrointestinal tract [27]. Tang et al. found that visual estimation is subject to both low intra-observer and inter-observer agreement for grading of gastrointestinal neuroendocrine tumors. Compared to manual counting (of all individual tumor cells), automated counting showed excellent concordance (98%) [27]. The usefulness of automated Ki-67 in predicting gastrointestinal neuroendocrine tumor has been clinically validated at least in one study [28].

The potential technical limitations of automated analysis of Ki-67 PI are: a requirement for in-house validation of the method against manual cell count; counting a sufficient number of tumor cells to avoid misrepresentation of Ki-67 from intra-tumoral variability; and methods to exclude counting on non-tumor mitotic activity. Regarding validation, the College of American Pathologists has issued guidance on validation of quantitative assays for estrogen receptor and progesterone receptor that are helpful in establishing standards for clinical validation [16]. Regarding cell counts, the WHO recommends counting at least 500 cells for grading neuroendocrine tumors of the GI tract; however, we tried to limit intra-tumoral variability by using a much higher threshold of 2000 tumor cells across at least 3 distinct manually annotated representative tumor fields. Automated image analysis renders scoring a large number of cells a trivial additional burden. The last issue, exclusion of non-tumor cells is the most complicated. The combination of manual selection of tumor fields with few non-tumor cells, and use of a counting system that uses size and/or other criteria to exclude intratumoral inflammatory cells is necessary to overcome this problem.

The current study reports similar results to previously published data on overall survival between LCNEC and carcinoid tumors. However, we did not find a difference in overall survival between TC and AC, probably due to relatively low number of AC (n = 7), a limitation in our study. One patient with AC in our study was unusual, in that death occurred 2.5 months after diagnosis, but this patient had delayed medical treatment and was initially diagnosed with distant metastasis. Our follow-up data did show a marked biologic difference between LCNEC and carcinoid tumors; all LCNEC with available follow-up data (8/8) recurred with a poor median overall survival of 19.7 months. There was a significant stepwise decrease in DFS with tumor grade, similar to previous studies; the reported 5 year survival rates are 87-97%, 56-78%, and 15%-40%, respectively for TC, AC, and LCNEC [6-8,12,21,29,30]. We found a strong association between black race, smoking and LCNEC, and in the latter, a traditional risk factor shared with small cell carcinoma [6,7]. In contrast to other series showing no gender predilection [1], we showed a predominance of women (3:1 ratio) for AC and TC.

## Conclusion

The current study identified cutoffs that reliably separated surgically resected endocrine tumors of the lung by Ki-67 proliferative indexing using automated digital image analysis. With our system, which has been validated for prognostication in breast cancers, a cutoff of 7% separated AC from TC, and there was a wide area (between 30 and 60%) that separated LNEC from AC.
